# Ultra-Low Power Optical Sensor for Xylophagous Insect Detection in Wood

**DOI:** 10.3390/s16111977

**Published:** 2016-11-23

**Authors:** Angel Perles, Ricardo Mercado, Juan V. Capella, Juan José Serrano

**Affiliations:** Instituto ITACA, Universitat Politècnica de València, Camino de Vera, s/n, 46022 Valencia, Spain; rmercado@itaca.upv.es (R.M.); jcapella@itaca.upv.es (J.V.C.); jserrano@itaca.upv.es (J.J.S.)

**Keywords:** termites, low-power, detection algorithms

## Abstract

The early detection of pests is key for the maintenance of high-value masterpieces and historical buildings made of wood. In this work, we the present detailed design of an ultra-low power sensor device that permits the continuous monitoring of the presence of termites and other xylophagous insects. The operating principle of the sensor is based on the variations of reflected light induced by the presence of termites, and specific processing algorithms that deal with the behavior of the electronics and the natural ageing of components. With a typical CR2032 lithium battery, the device lasts more than nine years, and is ideal for incorporation in more complex monitoring systems where maintenance tasks should be minimized.

## 1. Introduction

Xylophagous insects, especially termites, can cause structural damage to wood structures, including high-value historical buildings [1,2,3] and masterpieces. Collection care, which is sometimes called preventive conservation [4,5,6], can be greatly enhanced if automated continuous monitoring techniques can be applied in order to detect dangerous situations, such as moisture, extreme temperatures or pests. Automated techniques could simplify the effective implementation of vulnerability analysis [7].

There is a wide range of techniques for detecting pest [8,9,10,11,12,13] including acoustics, optical (infrared, visible light, laser, etc.), bait systems, machine vision systems, magnetic resonance imaging (MRI), thermal imaging, X-ray computed tomography, chemical chromatograph. Some of them require too costly equipment, so they should be discarded for massive production. In any case, the detection of insect activity using routine visual inspection has to be considered as an unreliable method [14].

Detection methods based on acoustic technologies have been proposed in the literature [15,16,17,18,19,20,21], including ultrasonic ones [22], and have been patented, but are unreliable due to external interferences, such as human behavior or structural vibrations, involving excessive false positives. Acoustic and piezoelectric techniques are adequate for some species, especially for scrapping, but it is nearly impossible to design an efficient autonomous electronic device due to the high energy requirements in the recording and processing of insect signals. However, the acoustic technique has been successfully applied in other scenarios [9,23] and we can expect an increased efficiency by means of the use of MEMS-based (Microelectromechanical systems) microphones.

Techniques for the detection of small insects, such as termites, prevent the use of conventional crossing detectors (systems based on a transmitter and receiver to detect a passing beam cut-off) [15].

Finally, Bait systems [24] are efficient for treatments, but imply a high logistic cost because they need to be open in order to monitor termite or other insect activity Bait techniques can be efficiently combined with others methods, including complex machine vision systems in order to automatically inspect the traps [8] and it is an important research approach in the area of precision agriculture.

A long life sensor without maintenance is crucial in order to apply it in real Internet of Things (IoT) [25] environments (smart homes, smart heritage, singular wood structures, etc.); furthermore, in this kind of application, its applications is necessary to reduce operator intervention. The size of the proposed sensor should be small and its design should be simple enough in order to facilitate its use.

In view of this, the development of a low-power electronic sensor to embed in wood will be presented. Among various investigated techniques, and after the necessary preliminary experiments, the authors propose the application of optical detection techniques, based on variations in the amount of absorbed/reflected light. The detection of insects is done using illumination LEDs and light sensors that read reflection variations when an insect such as termites, ants, cockroach, etc. approaches the device. 

In this paper, we present detailed design of an ultra-low power sensor to monitor wood masterpieces and structures of heritage buildings for detecting pests. We designed the sensing principles and implemented it for the enterprise AIDIMA (Furniture, Wood and Packaging Technology Institute) and has been successfully applied in monitoring different types of wood structures [24,26].

The design excels from the point of view of energy requirements, and results in an ultra-low design ideal for IoT applications. It can be associated with other complementary sensing techniques, including equilibrium moisture content in wood (EMC), temperature, pressure, acceleration, etc. The combined EMC techniques were presented in reference [24].

The article is structured as follows; In Section 2, the proposed sensor design will be presented, detailing all its features. In Section 3, the implemented detection algorithm will be explained, presenting the experimentation test results. This algorithm has been oriented in order to minimize factory (and subsequent) adjustments. In Section 4, experimentation and validation in “real” environments for the proposed sensor and detection algorithm will be presented, showing the results obtained. Finally, we present the main conclusions and future work in Section 5.

## 2. Sensor Design

**Figure 1:**
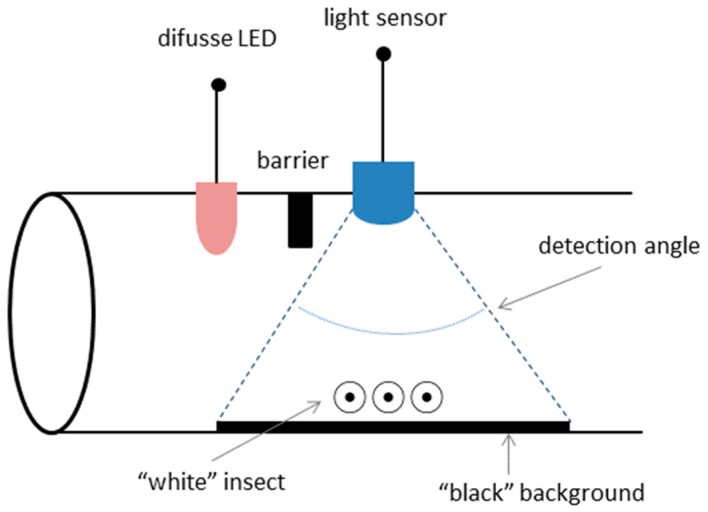
Proposed sensor diagram.

For the insect detection sensor development, it is proposed to use a hollow cylinder, in which insects enter (attracted by bait or something similar). Once inside, the detection principle uses optical techniques, based on variations in the amount of absorbed/reflected light. Figure 1 shows a diagram that summarizes the proposed principle.

For example, for its application on white termites (*Reticulitermes lucifugus*) we assume a “white” colored insect and a “black” container cylinder. Inside the cylinder, a source of “white” light and a photodetector to collect the light reflected on the tube are placed on the same side.

The amount of light received by the sensor will vary depending on the presence of termites, because they will reflect light. To detect the insects, the variations in reflected light must be evaluated along with other parameters, such as electronic inherent noise, ageing, dust, temperature effect, etc.

The choice of a photosensor with a fairly broad spectrum will allow to adapt to the color of the insect. For example, for a green insect, the light could be green. If it is black, the tube will be colored in white, etc.

**Figure 2:**
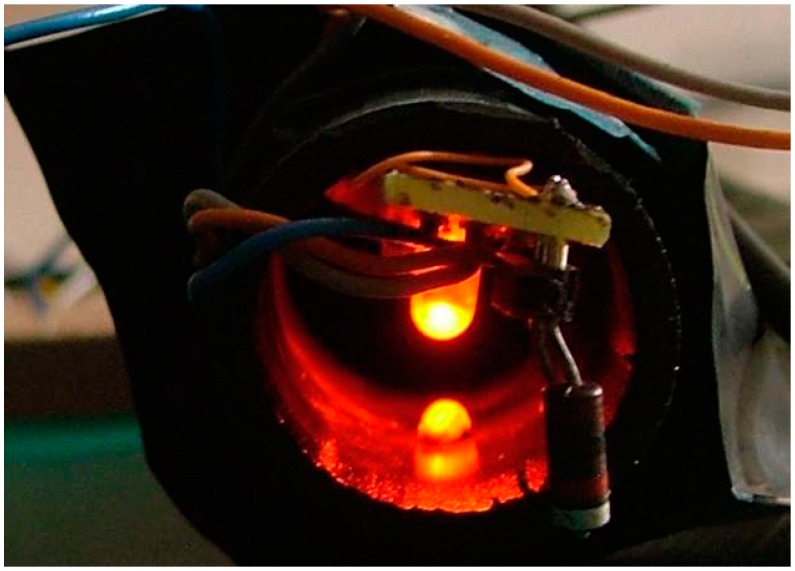
First operative experimental prototype of the proposed sensor.

Figure 2 shows the assembly made for initial tests. A TSL-250 light (AMS Ag., Premstaetten Austria) sensor with an integrated amplifier and an oval red E1L4E LED (Toyoda-Gosei, Inazawa, Japan) of high efficiency are used. The LED is oval in order to concentrate the light on one area, so that the insect crossing affects the light change more.

The initial board was mounted within black polyethylene tubes and, although different detectors have functioned well, the device shown in Figure 2 presents the best results.

Several experiments were carried out in the Institute for Information and Communication Technologies (ITACA) lab, with an arthropod inside the tube, obtaining variations of 0.1 V, which are adequate for implementation using a microcontroller and an AD converter, which allows analysis of the variations.

In line with this, the assembly was formed by an oval LED Toyoda-Gosei E1L4E and the TSL250 sensor was associated with a C8051F310 (Silabs, Austin, TX, USA) microcontroller. The obtained results are shown in Section 3.

**Figure 3:**
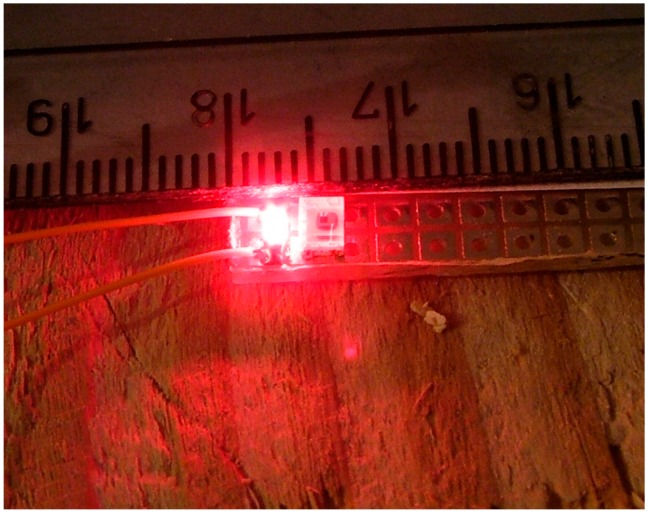
Proposed sensor’s final appearance.

Based on the results of the preliminary experiments, and taking into consideration that the final size of this type of sensor is a very important feature, a sensor based on surface mounted devices (SMD) components has been designed. The light sensor was replaced by a TSL-12T (AMS Ag., Premstaetten, Austria), which is slightly less sensitive, but has a wider angle of reception than the previous device, making it ideal for collecting information on more parts of insect passage. The illumination LED was replaced by a high efficiency wide angle HSMZ-A100 LED (Avago, San José, CA, USA). Finally, the detector size was set to 6 mm × 5 mm. Figure 3 shows the red LED emitter prototype in the SMD format and the TSL-12T sensor.

**Figure 4:**
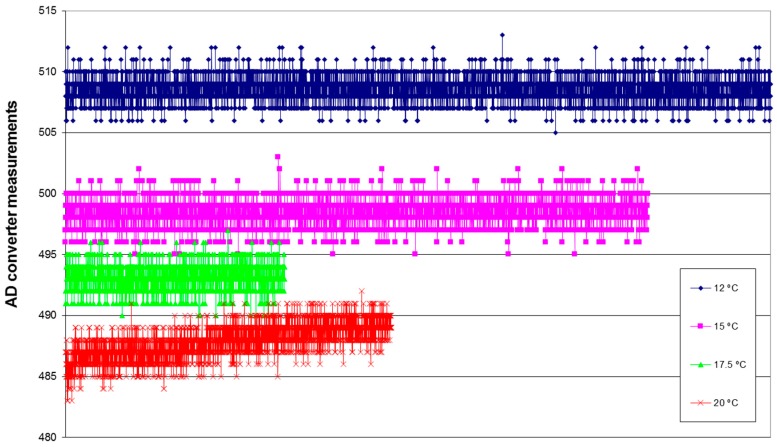
Temperature and noise effects in the detection system.

A main drawback of this design is the effect of temperature in the signal provided by the LED and collected by the light sensor. Figure 4 shows a set of readings obtained from the microcontroller AD converter for different ambient temperatures without the target object being present. Low noise is observed in the sensor. The AD converter has 10 bits and the voltage reference is 3.23 V. Obviously, the effect is due to the ensemble LED + sensor + “wrapper” tube + voltage reference (battery), etc., so the detection algorithm must take this all into account. The read voltage corresponds to a decrease of about 10 mV per degree Celsius, which should be taken into consideration when developing the detection algorithm.

To create an adequate detection algorithm, first we collected the measurements of the effect of the insects on the sensor. For this, the prototype was prepared to send acquisitions to a computer using a serial connection. For these collecting sessions, the microcontroller performed around 3.5 sampling per second.

The goal of this communication technique was only to evaluate the behavior of the detection algorithms, not the communication technologies utilized in the final device. In the final design, the node used wireless communications using the 868 MHz ISM band.

The sensor was covered at one end with black matte plastic and the system was put into operation to take samples. 

**Figure 5:**
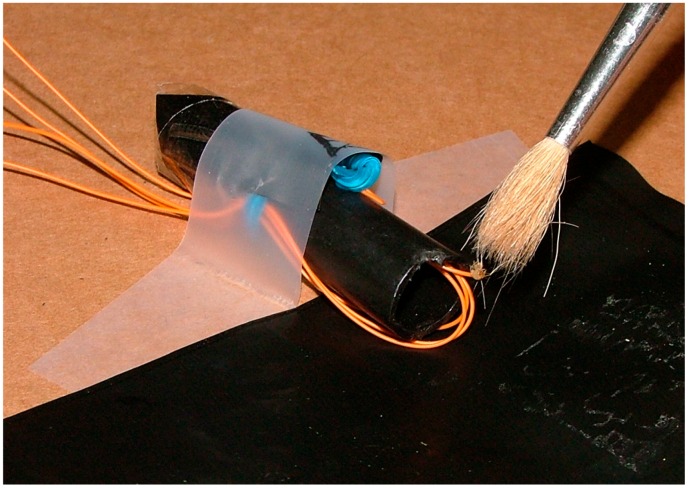
Termite being introduced into the detection sensor.

Several tests with different types of insects were carried out. For example, Figure 5 shows a termite being introduced into the tube by the entomologist.

**Figure 6:**
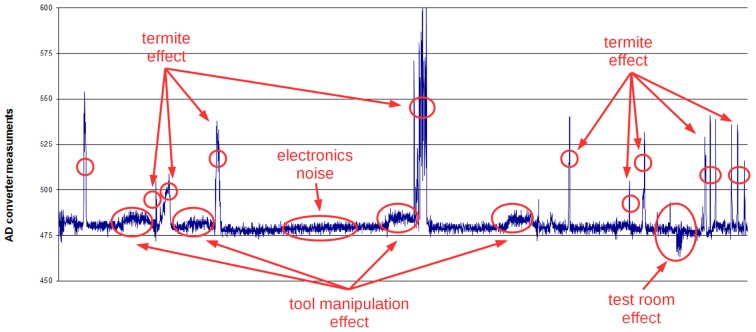
Effect of termite movements within the cylinder.

The graph shown in Figure 6 contains an example of the test session. In this figure, a typical background electronic noise added to the signal can be seen. Additionally, the effect of the light reflection on the body of the termites clearly affects the measurements. The effect of the tool utilized by the entomologist to introduce the termite can be seen in the graph as being non-negligible. All the experiments were performed in a dark room, and a simple open–close of the door is “seen” in the readings of the sensor. It can be observed that the proposed sensing system clearly “sees” the passing of the insect. The effect of tools and surrounding light promoted a change in the sensor design in order to put the detection electronics in the inner part of the sensor to avoid the effect of wood translucency.

## 3. Detection Algorithm

Our requirements for the algorithm were:

- Low power operation in order to maximize battery life.
- Minimum initial adjustments for self-tuning for dealing with variability in electronics and to reduce factory production costs.
- Near 0 false detections to avoid personnel displacement and undesired wood interventions. This problem is typical in acoustic/vibration-based systems.
- Maximum true positives.

To deal with these requirements, the algorithm has two phases of operation:

- Automatic adjustment: In which, during an initial period, samples from the sensor circuit are obtained in order to know its state.
- Motion analysis: In which, at fixed intervals, the LED emits radiation and the light sensor reads levels to detect whether there has been a change, within thresholds, in the reflection of this radiation.

The pseudocode of Algorithm 1 provides a simplified view of the operation of the algorithm that we named “minmax”, and Table 1 describes the configuration parameters. 

 **Algorithm 1.** Pseudocode of the detection algorithm. value = 0.0 for N do  value = value + sensor_sample_data  sleep SLEEP_TIME min_value = max_value = value / N samples_count = 0 always  value = sensor_sample_data  if value > max_value   max_value = value  if value < min_value   min_value = value  if absolute(max_value-min_value) > MINMAX_THRESHOLD   detection = true   max_value = min_value = value  else   detection = false  samples_count = samples_count + 1  if samples_count = FORGET_THRESHOLD   samples_count = 0   max_value = max_value -1   min_value = min_value +1  sleep SLEEP_TIME

The initialization phase averages a set of N samples and uses the mean value as an initial reference for the algorithm. This step deals with electronics ageing, dust, reference voltage, and initial temperature of the system.

The electronics sleep “SLEEP_TIME” between samples in order to reduce energy requirements. It can be safely established in two seconds in order to warrant termite detection during sensor crossing. Higher times save energy at the expenses of detections.

Then, the algorithm enters the motion analysis phase. In this phase, the sensor is read and compared with previous maximum and minimum readings. If the read value exceeds one of these parameters, then this value becomes the maximum of the minimum read.

A detection is triggered when the difference between the maximum and minimum exceeds a threshold (MINMAX_THRESHOLD). This threshold is critical for distinguishing between noise and true insects. An excessively high value provides an insensitive device, and values that are too small could provide false positives. A detection resets the difference between the maximum and minimum, so the algorithm is ready for detecting new variations.

Finally, the minimum and maximum values are approached every FORGET_THRESHOLD parameter measures in order to forget small variation effects of peaks of electronic noise. This mechanism allows to follow temperature drifts without triggering false positives. Taking into considerations that the sensor should run without intervention for years, this mechanism is also responsible for assuming the effects of ageing and dust.

Real traces based on data obtained with termites were used to check the operation of the proposed detection algorithm.

**Figure 7:**
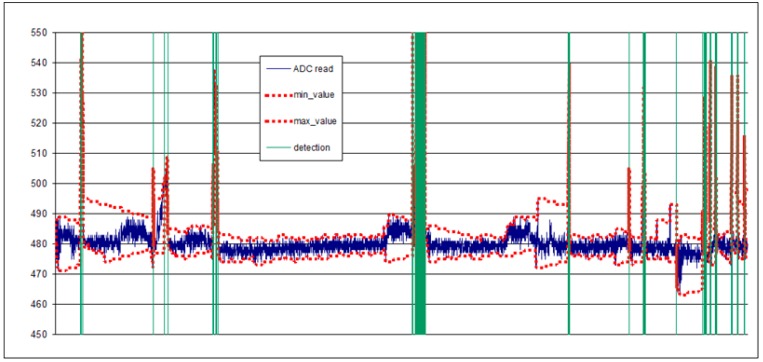
Proposed algorithm operation on a real trace.

Figure 7 corresponds to the data shown in Figure 6 after the application of the algorithm. As shown, the algorithm smoothly detects the changes caused by termites. By design, the algorithm is insensitive to noise and drifts, thanks to the included compensation mechanism.

In order to evaluate the goodness of the algorithm for false positives, it was implemented in the microcontroller and whole system was tested for 11 h, with the sensor placed inside a dark box and subjected to ambient temperature variations. The experiment did not generate any detections, providing 0 false detections. The next section shows a testbed, where a reference sensor without termites ran for six months without providing any false detections.

After these initial tests, the algorithm was tested exhaustively using all the collected real traces and was correlated with the expected results with controlled fake insects and real insects, obtaining high confidence results. Based on these data, we estimated 93% true positives, and the cause of non-detection was insects being very near the LED emitter so no light variations could be detected.

As a remarkable feature of the algorithm, we can mention that only a few parameters for adapting it to any insect are needed, and that it is insensitive to aging electronics and small accumulations of dirt on the lenses.

## 4. Experimentation: Results and Discussion

An exhaustive experimentation has been carried out in order to verify the practical operation of the algorithm under real conditions.

The first experiment consisted of mounting a sensor node in AIDIMA’s lab. The sensor node was placed in a pot with a termite colony (*Reticulitermes lucifugus*).

**Figure 8:**
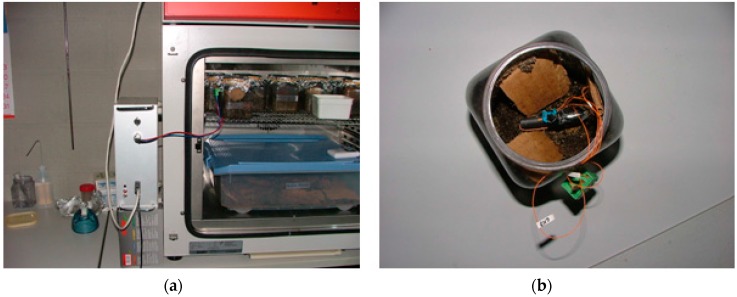
Experimental setup in the climatic chamber (**a**); Reference pot (**b**).

**Figure 9:**
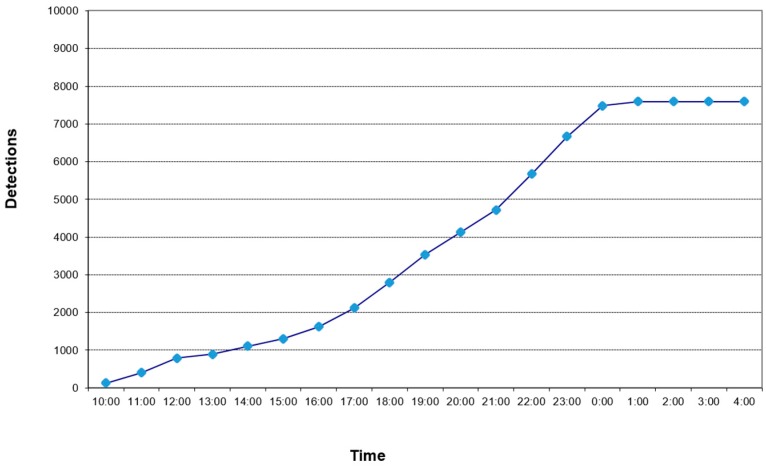
Cumulated registered detections.

The pot was placed in a climate chamber, as shown in Figure 8a, and the detection test started at 11:00 a.m. and detections cease at 2:00 a.m. Figure 9 shows cumulated detections for this experiment. The system check shows that termites built a mud trail that isolates them from the sensor. It is possible that termites tried to avoid the red light utilized by the detector, but others types of light, such infrared, could be applied [15].

**Figure 10:**
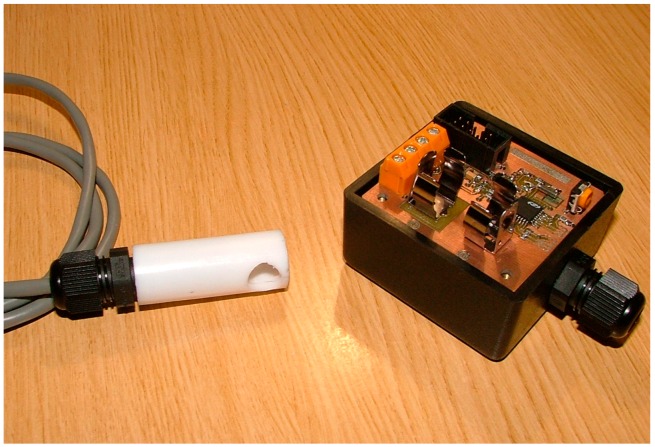
Developed node with the proposed sensor.

In the next experiment, five sensors nodes were mounted in order to check the operation of the proposed sensor device in a typical installation. Figure 10 shows the type of node mounted in this experiment; it uses an RS-485 connection to transmit the results to a collecting computer.

The assembly consisted on installing a total of five nodes on pieces of *Pinus radiata* wood. The piece is drilled in order to insert the sensor, and small holes are made in the bottom part of the piece to facilitate the entry of termites.

One of the nodes was mounted on a bucket with soil without termites, Figure 8a, and the rest of nodes were mounted in a box with soil and a termite colony. The entire system was introduced in the same climatic chamber shown in Figure 8a in order to maintain the necessary moisture conditions for termite life.

The nodes were interconnected and a cable was run from the chamber in order to carry the signal of the RS-485 bus to the computer in order to collect the data.

After six months of data collection, the installation was dismantled. The apparent state of the system was registered and results were analyzed.

The temperature data indicate stability between 25 °C and 27 °C. This stability is probably due to regulation of the chamber where the installation and experimentation were carried out.

**Figure 11:**
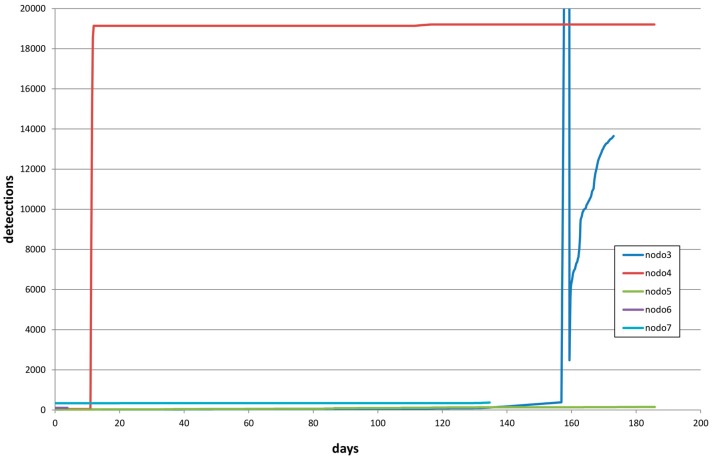
Detection counter evolution.

Concerning detection accounts, Figure 11 shows the evolution of the counter for each node.

**Figure 12:**
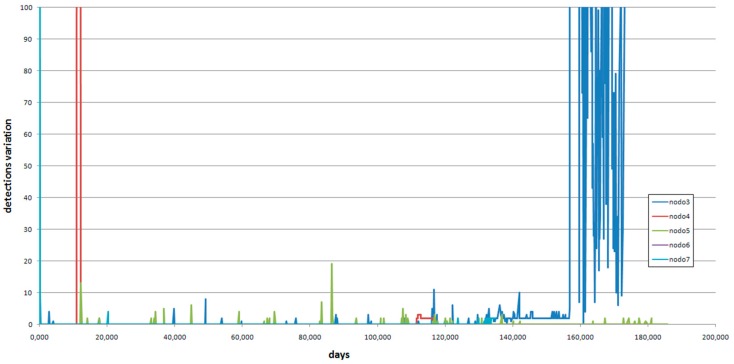
Instantaneous detection numbers.

It is difficult to determine the number of detections, so the graph in Figure 12 shows proposed instantaneous detections.

In node 3, variations are observed from approximately day 135, significantly increasing variations around day 155. These variations continue until the end of the available data collection.

A strong variation around day 10 is found at node 4, and minor variations are observed from that date.

In node 5, low activity is found, but not null.

For node 6, only data for 3 days are available.

In the node 7, barely any activity is detected.

**Figure 13:**
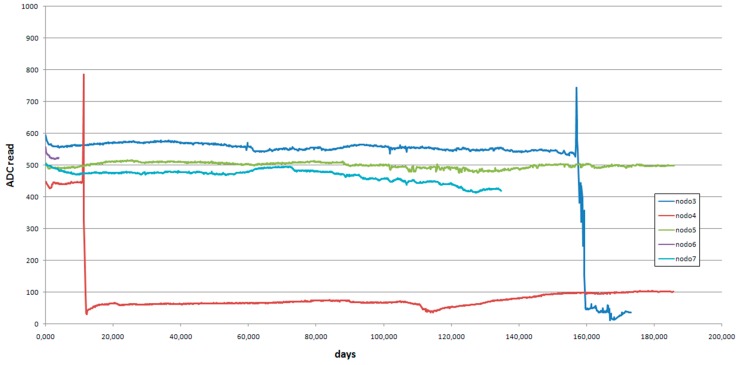
Recorded values using the light detector.

With the intention to have more information to understand Figure 12, the graph in Figure 13 was generated using the last value taken by the motion detector in each data transmission. The effect of mud trail creation in the value evolution is remarkable: A significant increase and fast reduction occurs.

**Figure 14:**
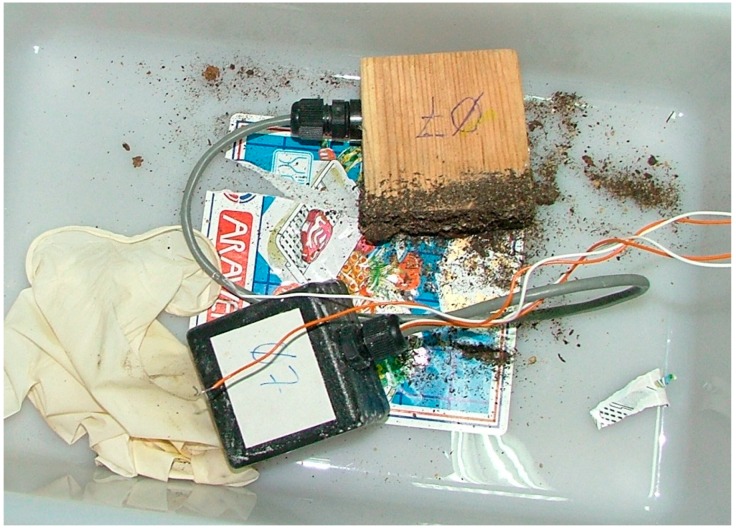
Sensor 07 appearance after the experiment.

The first node disassembled was identified with the “node 07” tag, and corresponds to the separate pot. Apparently, termites were not observed. Figure 14 shows the node–wood set extracted from the pot.

Then, node 05 was unmounted. A termite attack is found, but the sensor cylinder is not blocked. The sensor circuit is in good condition.

**Figure 15:**
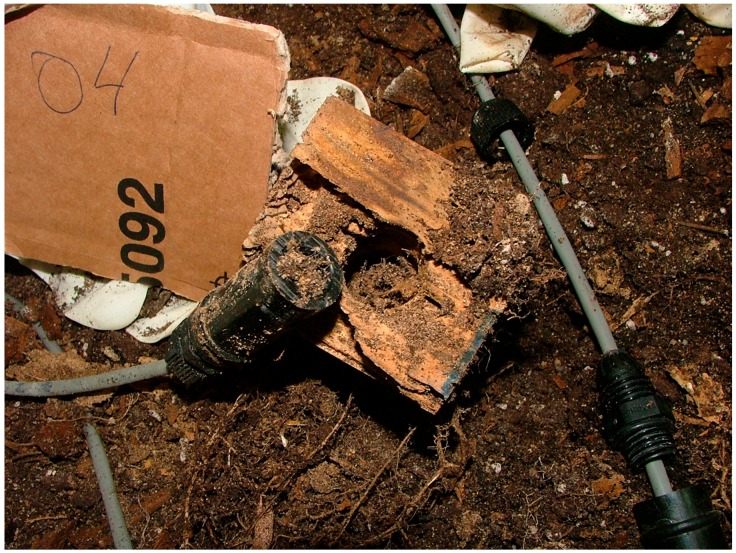
Sensor 04 appearance after experimentation.

The next node removed was node 04. A number of termites were observed inside, and the sensor was blocked. The piece of wood was very affected by termite attack. Figure 15 shows the appearance of the cylinder (full of soil) and the piece of wood where node 04 was inserted. 

**Figure 16:**
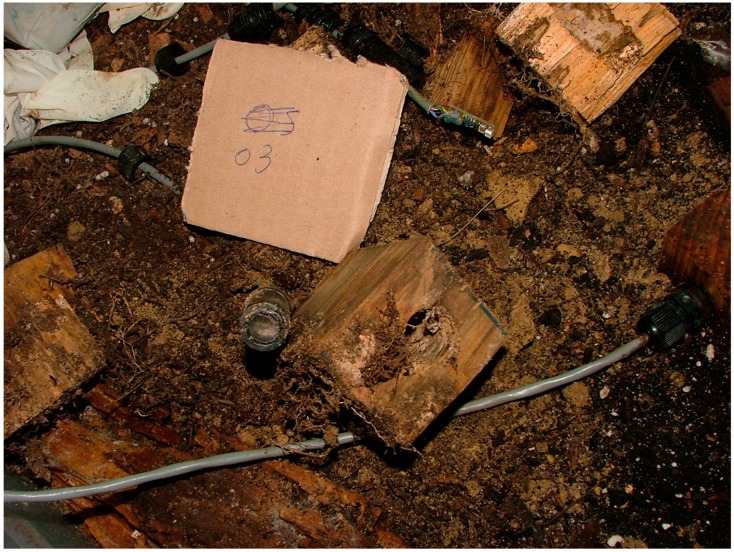
Sensor 03 appearance after experimentation.

The next node disassembled was node 03 and termites were observed inside the cylinder. The sensor was not blocked, but a cobweb of mold was observed. It was verified that the LED still worked and there was soil in the circuit. Figure 16 shows the appearance of node 03 after being disassembled.

Finally, node 06 was removed. Outwardly, the wood looked as if it had been attacked, but it did not seem that termites had reached the cylinder. The piece of wood was wet and the copper of the circuit was very oxidized. At the end of the experiment, this node stopped working; thus, the final design included tropicalization technology in order to avoid this issue.

## 5. Final Node and Energy Requirements

**Figure 17:**
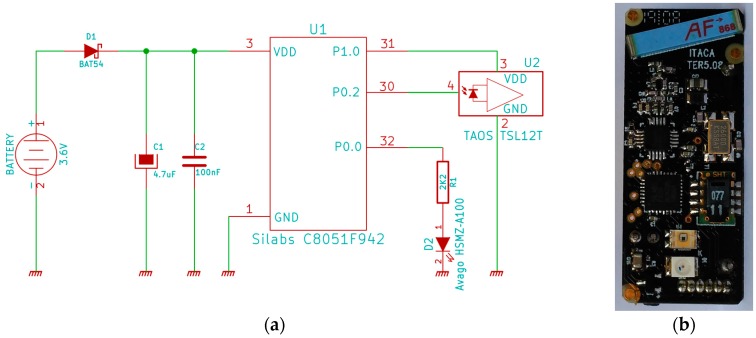
Electronic schematic of the sensor part (**a**) and electronic board implementation results (**b**).

With all these requirements and experiences, the final electronic design is shown in Figure 17a, where the microcontroller was replaced with an ultra-low power C8051F920 (Silabs, San José, CA, USA) microcontroller. Figure 17b shows an image of the board with these components and others, such as a moisture sensor and an ISM (industrial, scientific and medical) ISM 868 MHz radio; the light sensor and the diode are at the bottom of the board. We selected a thin black-coated electronic board in order to maximize the detection of the particular xylophagous insects.

Taking into account the very restricted energy requirements (we desired to produce a long-lived battery-operated device), both the LED and the light sensor cannot be driven continuously, thus, we decided to turn on these electronic elements for the necessary amount of time to prepare and to take an analog voltage measure. Our tests showed that 400 ms are enough for this particular electronic configuration, but we decided to select a less strict value of 1 ms in order to avoid the possible effects of other parameters (temperature, ageing, etc.). The rest of the time, the electronics can be turned off.

The energy aspects of the proposed sensor nodes are of capital importance in this type of systems, as a long autonomous operation is desired.

The energy requirements in Joules for each component of the proposed sensor can be calculated using Equation (1).

*E* = *I* × *V* × *t* (1)

where I is the current in amperes, V is the voltage in volts, and t is the time in seconds. 

In real implementations, the energy requirement of each electronic part depends on its state. It is fundamental to test each part, individually, in different configurations, in order to check whether the energy requirements specified on the datasheets are correct. For the calculations, the maximum energy consumption has been considered in order to have a worst-case scenario battery life estimation.

The purpose of this section is to analyze the real energy required by the proposed sensor. In order to determine the battery requirements, energy is measured in Ah (amperes × hour) and not in Joules; this is the unit capacity of batteries.

In this type of design, microcontroller selection is key for maintaining energy requirements as low as possible. Based on our initial tests, this was the reason for changing to a more energy-efficient option, based on the C8051F920 (Silabs, San José, CA, USA) microcontroller.

The maximum energy required for a full operating sensor is reflected in Table 2. This table includes the maximum energy for every part, and time, in each consumption mode.

The complete detection system requires only 17.6 mAh of energy per year. This low energy profile is obtained thanks to the reduced time involved in sensing (LED + light sensor) and the low energy requirements of the microcontroller when in sleep mode.

In order to maintain the energy requirements as low as possible, we only provide power to some electronics (e.g., the TAOS sensor) when measurements are required.

To provide a rough estimation of a battery-powered design using this technique, Table 3 shows the estimated maximum battery life for different battery models.

The results in Table 2 show excellent sensor lifespans, allowing the node to survive for a long time without requiring maintenance. As a typical reference, a CR2032 coin-cell battery can power the sensor for more than nine years, but higher energy batteries can be applied to provide better lifespans.

## 6. Conclusions 

The proposed pest sensor opens a wide field of possibilities and interesting applications in the sensing field for home automation and the protection of historical–artistic heritage.

The current formulation and configuration of the detection algorithm is sufficient to successfully detect activity and obstructions.

In our case, we included this sensor in production-ready wood with inserted pests, and we measured the equilibrium moisture content with the wireless sensor nodes.

Verifications were obtained when the installation was dismantled, which fit the results obtained from the collected data, which validated that the node operation was correct.

As for the duration of the nodes, it has been proven that the operation time exceeds the theoretical calculations. Therefore, the theoretical calculations can be considered to be correct.

## Figures and Tables

**Table 1:** Main parameters of the algorithm.

Parameter	Description
SLEEP_TIME	Time between samples. The sensor is kept in ultra-low power during this time.
MINMAX_THRESHOLD	Reference value utilized for deciding a detection.
FORGET_THRESHOLD	Number of samples interval to artificially reduce the measured difference between data readings.

**Table 2:** Energy requirements for each part. Power voltage = 3.3 V.

Description	Current (μA)	Working Time per Day (s)	Annual Requirements (mAh)
Microcontroller sleep + RTC	1.0	86,398.60	8.76
Microcontroller active	3000.0	2.0	0.61
LED active	1000.0	43.20	4.38
AMS-TAOS light sensor active	780.0	43.20	3.42
AMS-TAOS light sensor unpowered	0.0		0.0
		Total energy required (mAh)	17.16

**Table 3:** Estimated sensor battery life.

Battery Model	Capacity (mAh)	Usable (%)	Estimated Lifetime (years)
Panasonic CR2032	220	75	9.6
Lithium-thionyl 2/3 AA 3.6 V	1700	75	74.3
Duracell Ultra 3 v. 27 × 15.6 diam DCLR2	950	75	41.3
